# The intertwining of Zn-finger motifs and abiotic stress tolerance in plants: Current status and future prospects

**DOI:** 10.3389/fpls.2022.1083960

**Published:** 2023-01-04

**Authors:** Debojyoti Moulick, Karma Landup Bhutia, Sukamal Sarkar, Anirban Roy, Udit Nandan Mishra, Biswajit Pramanick, Sagar Maitra, Tanmoy Shankar, Swati Hazra, Milan Skalicky, Marian Brestic, Viliam Barek, Akbar Hossain

**Affiliations:** ^1^ Department of Environmental Science, University of Kalyani, Nadia, West Bengal, India; ^2^ Department of Agricultural Biotechnology & Molecular Breeding, College of Basic Science and Humanities, Dr. Rajendra Prasad Central Agricultural University, Samastipur, India; ^3^ School of Agriculture and Rural Development, Faculty Centre for Integrated Rural Development and Management (IRDM), Ramakrishna Mission Vivekananda Educational and Research Institute, Ramakrishna Mission Ashrama, Narendrapur, Kolkata, India; ^4^ Department of Crop Physiology and Biochemistry, Sri University, Cuttack, Odisha, India; ^5^ Department of Agronomy, Dr. Rajendra Prasad Central Agricultural University, PUSA, Samastipur, Bihar, India; ^6^ Department of Agronomy and Horticulture, University of Nebraska Lincoln, Scottsbluff, NE, United States; ^7^ Department of Agronomy and Agroforestry, Centurion University of Technology and Management, Paralakhemundi, Odisha, India; ^8^ School of Agricultural Sciences, Sharda University, Greater Noida, Uttar Pradesh, India; ^9^ Department of Botany and Plant Physiology, Faculty of Agrobiology, Food, and Natural Resources, Czech University of Life Sciences Prague, Prague, Czechia; ^10^ Institute of Plant and Environmental Sciences, Slovak University of Agriculture, Nitra, Slovakia; ^11^ Department of Water Resources and Environmental Engineering, Faculty of Horticulture and Landscape Engineering, Slovak University of Agriculture, Nitra, Slovakia; ^12^ Division of Agronomy, Bangladesh Wheat and Maize Research Institute, Dinajpur, Bangladesh

**Keywords:** abiotic stresses, Zn-finger proteins, mechanisms, plants, gene signalling ABA - abscisic acid

## Abstract

Environmental stresses such as drought, high salinity, and low temperature can adversely modulate the field crop’s ability by altering the morphological, physiological, and biochemical processes of the plants. It is estimated that about 50% + of the productivity of several crops is limited due to various types of abiotic stresses either presence alone or in combination (s). However, there are two ways plants can survive against these abiotic stresses; a) through management practices and b) through adaptive mechanisms to tolerate plants. These adaptive mechanisms of tolerant plants are mostly linked to their signalling transduction pathway, triggering the action of plant transcription factors and controlling the expression of various stress-regulated genes. In recent times, several studies found that Zn-finger motifs have a significant function during abiotic stress response in plants. In the first report, a wide range of Zn-binding motifs has been recognized and termed Zn-fingers. Since the zinc finger motifs regulate the function of stress-responsive genes. The Zn-finger was first reported as a repeated Zn-binding motif, comprising conserved cysteine (Cys) and histidine (His) ligands, in *Xenopus laevis* oocytes as a transcription factor (TF) IIIA (or TFIIIA). In the proteins where Zn^2+^ is mainly attached to amino acid residues and thus espousing a tetrahedral coordination geometry. The physical nature of Zn-proteins, defining the attraction of Zn-proteins for Zn^2+^, is crucial for having an in-depth knowledge of how a Zn^2+^ facilitates their characteristic function and how proteins control its mobility (intra and intercellular) as well as cellular availability. The current review summarized the concept, importance and mechanisms of Zn-finger motifs during abiotic stress response in plants.

## 1 Introduction

Crops usually encounter a wide range of hostile climatic fluctuations during their life cycles. Such abnormal environmental fluctuations are covered by stressors of both biotic origins, including infection by pathogens (virus, bacteria, fungi, etc.), attack by weeds, and insects, as well as by abiotic components also (Moulick et al., 2018d; Ghosh et al., 2020a; Ghosh et al., 2020b; Ghosh et al., 2021; Ghosh et al., 2022a; Ghosh et al., 2022b; Mateos Fernández et al., 2022; Tonnang et al., 2022; Jain et al., 2022a; Jain et al., 2022b). Among the abiotic stress including heat and chilling stress (Aslam et al., 2022; Haider et al., 2022; Ullah et al., 2022; Verma et al., 2022), limitation of water (drought), limitation of nutrients, elevated levels of salt, and hazardous/toxic metals and metalloids in the soil (Hernandez et al., 2000; Moulick et al., 2019; Saha et al., 2019; Sahoo et al., 2019; Moulick et al., 2021; Choudhury et al., 2022a; Choudhury et al., 2022b; Choudhury and Moulick, 2022; Mazumder et al., 2022).

Environmental stress can adversely modulate the field crop’s ability to maintain its yield potential, i.e., their determining yield despite satisfactory inputs and other factors. A field crop/plant’s vulnerability to adverse deviations from (maximum yield) yield potential is usually calculated by measuring the respective crop’s yield stability (Bhadra et al., 2021). The difference between the actual yield and yield potential of a particular site (agro-environment) is regarded as the yield gap (Mueller et al., 2012). Crops seldom touch their real yield potential in most broad-acre agri-environmental systems due to experiencing stress (s) during crop’s life cycle. Stress-affected plants exhibited three primary response segments: first is the alarm or apprehension phase, sometimes also called the initiation of stress; the next phase, i.e., the second phase is characterized by resistance or fight phase through the initiation of defence systems; and the last or third phase is the exhaustion phase or collapse stage where loss due to stress is evident (Larcher, 2003; Moulick et al., 2019; Sahoo et al., 2019).

Among the stresses, salinity (high Na^+^) is the critical agro-environmental factor that limits growth and yield/productivity. Under salinity stress, crops usually modulate their physiological processes by endorsing water acquisition and retention, and shifting the ion homeostasis management process (Parida and Das, 2005; Barman et al., 2022; Roy et al., 2022). At the same time, scarcity of available water to plants or drought stress results in decreased survival of plants, growth, and development due to metabolic imbalances. Drought is often linked with a lack of accessibility of groundwater in the land/soil but can also be worsened by greater evapotranspiration (Jaleel et al., 2009; Choudhury et al., 2022c; Jadhav et al., 2022). Such stress (drought) may occur under dry/humid conditions and with elevated air temperatures. The disparity in the water loss due to evapotranspiration flux and water uptake from soil may attribute to the key reason behind imposing drought stress (Lipiec et al., 2013; Dash et al., 2022; Sagar et al., 2022). On the other hand, toxic heavy metals and metalloids are also posing a serious threat to achieving agricultural sustainability in crop production with significant accumulation in the edible parts imposing a menace to the food chain (Moulick et al., 2016a; Moulick et al., 2016b; Moulick et al., 2018a; Moulick et al., 2018b; Moulick et al., 2018c; Saha et al., 2019; Chowardhara et al., 2019a; Chowardhara et al., 2019b; Saha et al., 2022).

From a genetic point of view, stress is a set of certain environmental conditions that prevent a crop from experiencing its complete genetic expression. An abiotic component-induced stress, not only due to the exchanges (mainly with signalling) with other organisms and that can negatively impact particular organisms in an agro-environment, is regarded as abiotic stress. The effect of abiotic stresses on the agro-environmental sector is a crucial threat presently intensified by anthropogenic activities and global warming (Kang et al., 2017; Mukherjee and Hazra, 2022).

Reports indicate that abiotic stresses not only impose adverse on a crop’s anatomy, physiology, biochemistry and but subsequently limit essential metabolic processes like respiration, photosynthesis, and growth when lingered for a long time often inducing death also (Hirayama and Shinozaki, 2010; Lima et al., 2015; Basu et al., 2016). Plants are equipped with mechanisms (physiological and metabolic) that may be crucial in lightening/mitigating agro-environmental stresses. These stress-induced shuffling crops’ metabolic machinery is controlled using the initiation of genetic networks or pathways. The outcome of this genetic alternation is imparting greater/better tolerance or resistance to certain stress (s) (Claeys and Inzéand Inzé, 2013; Thorpe et al., 2013). Upon exposure to environmental stresses, dangerous by-products to crop health were found to be instrumental to plants’ normal health and wellbeing. H_2_O_2_ (hydrogen peroxide), superoxide radicals, OH-radicals (hydroxyl radicals), are regarded as reactive oxygen species (ROS), generated as a result of leakage of electrons that occurs during the photorespiration and photosynthesis process (Thorpe et al., 2013; Kao, 2017; Moulick et al., 2021). Within the plant cells, the proper antioxidant defense machinery and ROS build-up sustain a steady-state balance (Hasanuzzaman et al., 2012). Keeping a satisfactory level of ROS within the cell permits adequate operation of redox biology (metabolic reactions) and the management of numerous phycio-biochemical processes vital for plant’s growth and development. This kind of harmony among ROS formation and ROS quenching is an example of intermediate level of ROS management/homeostasis (Hasanuzzaman et al., 2019).

These reduced oxygen radicals or ROS were reported to adversely influence key components of a crop’s metabolic cycle resulting in significant damage to cellular processes and death (Nouman et al., 2014). In order to lessen the excessive ROS production and subsequent oxidative stress, plants/crops have well-maintained anti-oxidative machinery that consists of non-enzymatic as well as an enzymatic unit that can bring equilibrium among ROS generation and quenching and protection of cellular damage even PCD or programed cell death also (Raja et al., 2017; Duan et al., 2012; Das and Roychoudhury, 2014; Choudhury et al., 2021a; Hossain et al., 2021).

The magnitude of ROS induced damage to biomolecules subjected to many factors like the concentration of target biomolecule(s), site of the particular biomolecule(s) in relation to the ROS production site, the rate constant of the reaction among biomolecules and ROS, efficacy ROS quenching components are to name a few (Davies, 2005). Increasing the antioxidant level within the plant cells can be achieved by spontaneously through use of genetic engineering or by supplementing can boost up the defense system of the plant and saving them from the adverse impact of ROS generated as a result of environmental stress (Mishra et al., 2006; Gupta et al., 2009; Kaur et al., 2022; Upadhyay et al., 2022). SOD (superoxide dismutase), CAT (catalase), and POX or peroxides are well-recognized enzymes involved in antioxidant systems that adjust the ROS homeostasis by reducing OH• into H_2_O_2_ of crops (Gill and Tuteja, 2010; Nouman et al., 2016). Whereas, in coordination, with enzymatic components, the non-enzymatic units of the antioxidant system (glutathione, flavanoid, lipids carotenoids, etc.) work on H_2_O_2_ through various means (Duan et al., 2012; Yin et al., 2020; Choudhury et al., 2022c).

At present, the main concern for the researchers working on developing suitable strategies to mitigate abiotic stress in crops, the main challenge is the complexities regarding stressor (s), i.e., abiotic components and the responses by crops towards the stressors. Apart from agronomic practices, irrigation management comparatively newer avenues like seed priming technology (Moulick et al., 2016a; Moulick et al., 2017; Moulick et al., 2018a; Moulick et al., 2018b; Moulick et al., 2018c), potentials of wild relatives (Hossain et al., 2022) potentials of metabolomics and next generation sequences are to name a few (Choudhury et al., 2021b; Hossain et al., 2021).In order to properly understand how stressors and crops interact at the molecular level, i.e. replication, transcription and translation. One such exciting topic of how the information imposed upon exposure to a stressor communicates at the cellular level is to illustrate the contributions made by transcription factors or Tfs. Among the well-known TFs, Zn-Fingers are of prime interest.

The Zn-finger was first reported as a repeated Zn-binding motif, comprising conserved cysteine (Cys) and histidine (His) ligands, in *Xenopus laevis* oocytes as a transcription factor (TF) IIIA (TFIIIA) (Miller et al., 1985). Since its first report, a wide range of Zn-binding motifs has been recognized and termed Zn-fingers. To meet the cellular demand, many proteins employ non-protein (often metallic ions) as cofactors. Among the metallic ions considered cofactors, transition metal ions are the most important due to their significant influence on modulating a wide range of cellular activities. From the periodic table’s perspective, d-block elements are found to be more actively involved as cofactors. Zn is one cofactor that can influence as much as >10% of human proteins with a pronounced impact on structural and catalytic activities (Andreini et al., 2006; Maret and Li, 2009; Kochańczyk et al., 2015). In the proteins where Zn^2+^ is mostly attached to amino acid residues and thus espousing a tetrahedral coordination geometry. The physical nature of Zn-proteins, defining the attraction of Zn-proteins for Zn2+is crucial for knowing how a Zn^2+^ facilitates their characteristic function and how proteins control its mobility (intra and intercellular) as well as cellular availability. In the mammalian genomes, encoded Zn-finger proteins dominate in number and are known as TF-regulators. A specific number and variety within Zn-finger or Zn-containing domains contribute to different cellular processes like regulation of transcription, binding to nucleic acid, and folding of proteins. Intermolecular attachment spots are the most significant and sort after when compared with similar intermolecular bindings. Due to having structural complexities (peptide chain composition, orientation, etc.), the intermolecular attachment of ligands and their respective targets poses serious challenges to analysis analyze (Maret et al., 2004; Kochańczyk et al., 2016). Findings have shown that an optimum level of Zn^2+^ concentration is essential to maintaining the stability and folding of protein subunits and for satisfactory performance of the catalytic activity of a particular enzyme (Keilin and Mann, 1940; Vallee and Neurath, 1954; Parraga et al., 1988). This innovative concept of small Zn^2+^-stabilized domains was further supported by the in-depth analysis of the TFIIIA sequence, which exhibited that a continuous stretch of nine tandemly repeated 30 amino acid residues (13 to 276) having two invariant pairs of Cys along with His residues coordinating one Zn^2+^. This particular pattern was later coined as ZF/zinc finger (Miller et al., 1985; Fairall et al., 1986; Rhodes and Klug, 1986).

In this review, we are going to assess the contributions made by ZFs in elaborating and imparting abiotic stress tolerance in field crops with unique references to salinity, water stress, thermos-stress, heavy metals, irradiation and elevated CO_2_ levels.

## 2 Zn-finger acellular perspective

### 2.1 Sub-cellular localization of Zinc Finger Proteins

Zinc Finger Proteins (ZFPs) contain a highly conserved signature domain consisting of 20–30 amino acid residues having the consensus sequence of CX2–4CX3FX5LX2HX3–5H (X denotes any amino acid). Structural differences in different ZFPs are basically due to the differences in positions and numbers of cysteine (Cys) and histidine (His) residues that interacts and bind to the zinc ion and are constituted of several sub-groups which are designated as Cys4/C4 (GATA-1), Cys6/C6 (GAL4), Cys8/C8, Cys2HisCys/C2HC (Retroviral nucleocapsid), Cys2His2/C2H2 (TFIIIA), Cys2HisCys5/C2HC5 (LIM domain), Cys3His/C3H/CCCH, Cys3HisCys4/C3HC4 (RING finger) and Cys4HisCys3/C4HC3 (Requium), DnaJ-like zinc finger protein and many others (Lyu and Cao, 2018; Han et al., 2020; Yuce and Ozkan, 2020; Ali et al., 2022). As ZFPs are basically the members of the most prominent transcription factor family having a DNA binding domain, they are generally localized to the nucleus after translation. However, the zinc finger domain, an important structural motif, is also reported to be involved in other cellular activities such as RNA binding, membrane association and protein-protein recognitions and interaction (Han et al., 2020). Thus, different types of ZFPs are localized to cellular compartments as per their specific role in cellular activities. Protein sorting in a cell can also offer clues about the functions of these proteins (Wang et al., 2020).

Among all the sub-groups of ZFPs, the C_2_H_2_ (TFIIIA) has been extensively studied in plants. It represents the large proportions of ZFPs in plants where 189 members in rice (Agarwal et al., 2007), 321 in Soybean (Yuan et al., 2018), 122 in *Cucumis sativus* (Yin et al., 2020) and many more members in other crops have been reported. Its DNA-binding motif is one of the best-characterized motifs having two residues each of Cys and His amino acids tetrahedrally combined into a zinc ion (Xie et al., 2019). Focussing on plant-specific C_2_H_2_ type ZFPs (Q-type C_2_H_2_-ZFPs), it is reported to have different lengths of long spacer between two Zn finger motifs as compared to other eukaryotic organisms. Apart from the Zn finger motif, the core sequence KXKRSKRXR, which is present in the N-terminal of protein sequences, acts as an NLS for sorting of C_2_H_2_ type ZFPs to the nucleus (Xie et al., 2019) and the QALGGH motif present in the helical region is required for binding to DNA (Kielbowicz-Matuk, 2012). Having multiple functions in the nucleus, most of the reported C_2_H_2_-type ZFPs in plants were found to be localized in the nucleus. Several experiments were conducted with the C_2_H_2_ type ZFP genes to confirm the nuclear localization, such as FEMU2 of *Chlamydomonas reinhardtii*, fused with the β-glucuronidase (GUS) reporter gene was bombarded into onion epidermal cell and found that FEMU2 protein was in fact localized to the nucleus. A similar experiment was conducted with *JcZFP8* gene of *Jatropha curcas* fused with the GFP reporter gene under the control of the CaMV35S promoter. The 35Sp : *JcZFP8:GFP* gene construct was injected into tobacco protoplast for transient expression, and the fluorescence signal results clearly showed the localization of 35Sp : JcZFP8:GFP fusion protein into the nucleus of the tobacco cells (Shi et al., 2018). WRKYs with its conserved consensus sequence of WRKYGQK along with zinc-finger-like motifs of C_2_H_2_ and C_2_HC type ZFPs also have a NLS for their localization to the nucleus (Bakshi and Oelmüller, 2014). In the nucleus, it acts as a transcription factor and binds to the TTGAC(C/T) W-box cis-element in the promoter of their target genes (Bakshi and Oelmüller, 2014; Chen W et al., 2019) and transcriptionally regulates the expression of target genes (Cheng et al., 2017).

Similarly, another extensively studied sub-class of plant ZFPs is Cys3His/C3H/CCCH type. Plant genome encodes large numbers of CCCH type ZFPs and has been identified and characterized in crop plants such as Chickpea (Pradhan et al., 2017), *Brassica rapa* (Pi et al., 2018) and many other plants. The CCCH-type ZFPs of plants have conserved CCCH motifs ranging from one to six copies, with the most prevalent consensus sequence of the C-X7–8-C-X5-C-X3-H motif in the middle of the protein sequence (Pi et al., 2018; Chen et al., 2020). Although C-X7–8-C-X5-C-X3-H is the most common motif in CCCH-type ZFPs in plants, other structural variations in the consensus sequences suggest different cellular localisation patterns and roles in cellular activities (Han et al., 2020). Many of the CCCH-type ZFPs are localized to the nucleus, such as AtZFP1, KHZ1 and KHZ2 of Arabidopsis (Han et al., 2014), SAW1 and OsC3H10 of rice (Wang et al., 2020; Seong et al., 2020). CCCH-type ZFPs such as AtTZF2/3, ZFP36L3 and ZC3H12a are localized to the cytoplasm [84-86), where some of the CCCH-type ZFPs, namely OsC3H10, AtTZF4-6, etc. gets co-localized with stress granules (SGs) and processing bodies (PBs) (Seong et al., 2020). Likewise, some CCCH-type ZFPs are localized to plasma membranes such as AtOZF1and AtOZF2 (Oxidation-related Zinc Finger 1) of Arabidopsis and PeC3H74 of *Moso bamboo* (Huang TL et al., 2012; Chen et al., 2020) where some of the members are involved in secondary wall synthesis in response to biotic and abiotic stresses (Zhang et al., 2018; Chen et al., 2020), while several other members of CCCH type ZFPs such as OsLIC of rice, AtTZF of Arabidopsis shuttle between the nucleus and the cytoplasm (Wang et al., 2008; Bogamuwa and Jang, 2013). Shuttling of CCCH type ZFPs between cytoplasm and nucleus is due to the presence of leucine-rich NESs (Nuclear Export Signals) and NLSs (Nuclear Localization Signals) which are mainly present in their N- or C- terminal of the protein sequence (Han et al., 2020). These shuttle signals are current in several CCCH-type ZFPs across the crop species indicating their potential role in stress responses and signal transduction (Wang et al., 2008; Chai et al., 2012). Sub-cellular localization to cytoplasm and membrane was observed in another class of ZFPs called RING ZFPs. Arabidopsis RING ZFPs, *AtRZFP* fused with *GFP* under the control of 35S promoter was transformed into onion epidermal cells, and the signal of *AtRZFP*-*GFP* was clearly observed in the cytoplasm and plasma membrane (Zang et al., 2016). Some of the RING ZFPs located in the plasma membrane and cytoplasm include *AtAIRP1, RHA2a*, *AtATL78* of Arabidopsis, *OsRDCP1, OsSIRH2-14, OsRFPv6* of rice, *LjCZF1* of *Lotus japonicas*, VpRH2 of grape are located to the plasma membrane (Han et al., 2021) and *ZmXerico2* of maize (Gao et al., 2012), *OsSIRH2-14* and *OsSIRP1* of rice (Hwang et al., 2016), *AtAIRP4, EMR* of Arabidopsis (Park et al., 2018), *CaDSR1, CaASRF1* of pepper (Lim et al., 2018; Joo et al., 2019) are located in the cytoplasm. Other studies suggested that other than plasma membrane and cytoplasm, RING ZFPs are also localized to nucleus and other cellular compartments. For instance, *CaASRF1, CaAIRF1, CaDSR1* of pepper (Lim et al., 2017), *AtHOS1, AtATRF1* of Arabidopsis (Tian et al., 2015; Kim et al., 2017; Qin et al., 2017) and *OsSADR1* of rice (Park et al., 2018) were located to nucleus, whereas, *OsSIRH2-14* of rice was not only located in the cytoplasm and plasma membrane but was also found to be localized to Golgi bodies (Park et al., 2019). Likewise, wheat *TaDIS1* was also reported to be localized to Golgi bodies (Lv et al., 2020). Rice RING-H2 zinc finger proteins *OsHCI-1* and *OsMAR-1* were found to be localized to the cytoskeleton particularly in microtubules (Lim et al., 2013; Park et al., 2018) and RING-H2 ZFPs of wild tomato *SpRING* was found to be localized to the endoplasmic reticulum.

GATA-1 which is one of the sub-group members of ZFPs conserved families of transcription factors regulating the expression genes involved in cellular processes (Zhang et al., 2015). With the consensus sequence of CX2CX17−20CX2C along with DNA binding domain, it binds to the WGATAR (W = T/A, R = G/A) sequence in the promoter region of the target genes (Behringer and Schwechheimer, 2015; Gupta et al., 2017). As a transcription factor family, it has to be localized to the nucleus for its activities and to confirm it a study was conducted using the GATA gene of Poplar (*P. deltoids*) where Arabidopsis plant was transformed with *PdGNC-GFP* gene fusion under the control of CaMV35S promoter. Its nuclear localization was confirmed as the 35S: *PdGNC-GFP* fusion protein was detected in the nucleus (An et al., 2014).

Likewise, *Brachpodium distachyon BdGATA13-eGFP* gene fusion under the control of 35S promoter which was used for the transformation of tobacco leaves was found to be localized into the nucleus (Guo et al., 2021). However, reports on *FIP* (FtsH5 Interacting Protein), which is a type of GATA-1 ZFPs highlighted its localization to plastid as well specifically to thylakoid membrane in response to abiotic stress signals (Lopes et al., 2018). Similarly, DnaJ-like ZFPs with their characteristic C-terminal tandem 4× repeats of the CxxCxxxG are reported to have roles in the accumulation of carotenoids in plastids of non-pigmented tissues (Osorio, 2019) and inducing chromoplast biogenesis and simultaneously repressing the chloroplast biogenesis and chlorophyll biosynthesis in the nucleus of de-etiolating cotyledons cells (Sun et al., 2019). With its functions specific to the nucleus and chloroplast, *DnaJ-like ZFPs are localized to both the nu*cleus and chloroplast. Chloroplast localization of DnaJ like ZFPs is due to the presence of N-terminal chloroplast transit peptide (cTP) along with C-terminal zinc finger domain (ZF) which is separated by two trans-membrane domains (TMs) (Chen et al., 2021a). For nuclear localization, ubiquitination of lysine58 in ORANGE/OR (a type of DnaJ like ZFPs) by UBC19 was reported to be essential to generate truncated OR ZFPs proteins (Chen et al., 2021a). Sub-cellular localization studies of another class of ZFPs called as FCS-like ZFPs (FLZs) were conducted as it was found to be active both in cytoplasm and nucleus. FCS-like ZFPs are reported to act as scaffold proteins for plant-specific SnRK1 complex which is involved in various stress responses and are reported to be localized to both cytoplasm and the nucleus in plants like Arabidopsis and Maize (Jamsheer et al., 2018a; Chen et al., 2021b). The distribution of ZFPs to different compartments of plant cell clearly indicates that this class of proteins are involved in several cellular processes either at both transcriptional and post-transcriptional level.

### 2.2 Transcription and post-transcriptional roles of Zn finger proteins

Many ZFPs acts as critical transcriptional regulators that correlate with their localization into the nucleus. However, it also interacts with RNA and other proteins to regulate the post- transcriptional expression of the target genes at RNA and protein levels, respectively (Han et al., 2020). As a transcription factor, it binds to the cis-acting element and subsequently activates or represses the expression of downstream target genes. The conserved zinc finger structure helps it bind to DNA double helix at specific sites to act as a transcription factor (Han et al., 2021). In plants like Arabidopsis, durum wheat and rice, a highly conserved sequence QALGGH of Q-type C2H2-ZFPs provide the ability for ZFPs to recognize the target sites and to further regulate the expression of downstream genes through activator or repressor domain (Lyu and Cao, 2018; Xie et al., 2019). However, the QALGGH sequence is not the only key and ubiquitous sequence for binding to target genes (Liu et al., 2022), the C_2_H_2_ type ZFPs may bind to the target site of the genes through DNA binding domain which is present in long spacers between the two adjacent zinc finger motifs (Sakamoto et al., 2004).

Apart from binding to DNA as a transcription factor, C_2_H_2_ ZFPs can also bind to RNA based on their bases and folding backbones, which recognize variants of phosphoric acid skeletons in RNA (Lin and Lin, 2018; Han et al., 2020). It is found that amino acid residues of C_2_H_2_ zinc finger proteins positioned at -1 and +2 of the a-helix play a vital role in its binding to RNA (Han et al., 2020). Upon binding to RNA, some of the members of ZFPs such as cleavage and polyadenylation specificity factor 30 (CPSF30) belonging to CCCH/Cys3His/C3H type ZFPs are involved in the polyadenylation step of pre-mRNA processing after forming a complex which is collectively called as CPSF (Shimberg et al., 2016). The RNA binding *AtCPSF30* known for involvement in the polyadenylation step of pre-mRNA also interacts with other molecules like calmodulin, however, its RNA binding activity gets reduced in presence of its other interacting molecules like calmodulin (Lee et al., 2012). For interacting with other proteins including other zinc finger proteins, ZFPs like the C2H2 type utilize domains such as L-box motif and EAR motif for interaction resulting in binding/prevention of binding of the target protein to DNA which in turn regulates the expression of downstream genes (Gamsjaeger et al., 2007; Brayer and Segal, 2008). Among the two motifs, the EAR motif which is the smallest known repressor domain in plants is reported to be essential for the inhibition of transcriptional activities (Hiratsu et al., 2004).

Correspondingly, WRKY proteins which are the type of Cys(2)-His(2) (C2H2) or Cys(2)-HisCys (C2HC) ZFPs, have two highly conserved domains with WRKYGQK sequences of WRKY domain and C-terminal zinc finger domain (Rushton et al., 2010). The target DNA binding site generally recognizes W-box cis-elements in the promoter region of target genes. For instance, a WRKY protein of *Hylocereus polyrhizus HpWRKY44* was found to be directly binding to the W-box element present in the promoter of *HpCytP450-like1* and transcriptionally activated the *HpCytP450-like1*, resulting into induced betalain biosynthesis in pitaya fruit (Cheng et al., 2017). However, some WRKY proteins bind to the promoter region other than W-box cis-elements (Chen et al., 2019). The selective binding of different WRKY TF members to W-box cis-element is based on neighboring DNA sequences outside of the W-box core motif (Bakshi and Oelmüller, 2014). Several experiments highlighted the role of the WRKY gene family to be associated with the regulation of transcriptional reprogramming in response to environmental stresses. For example, *PROPER* genes of Arabidopsis which encodes for small peptides which act as molecular patterns associated with injury or damage are perceived by *PEPR1* and *PEPR2* (leucine-rich repeat receptor kinases) and amplify the defense responses. Upon receiving the stress signal WRKY TF binds to the promoter of these two kinases and regulates their expression (Logemann et al., 2013).

Advancement in molecular techniques has helped to characterize various TF and regulatory element functions on a genome-wide scale, such as the utilization of the DAP-seq technique to discover the binding sites of TFs in DNA (O’Malley et al., 2016). Similarly, transcriptional role and binding sites of ZFPs like *AtWRKY33* under biotic were successfully identified using the techniques like ChIP-seq and revealed that *AtWRKY33* negatively regulates the *NCED3* and *NCED5* (ABA biosynthesis genes) to impart resistance against biotic stress like necrotrophic fungus (Liu et al., 2015). Some C2H2 type ZFPs are reported to transcriptionally regulate the expression of genes involved in programme cell death of the plants, thereby inducing PCD of plant cells (Yin et al., 2020).

Similar to C_2_H_2_ type ZFPs, many C_3_H/CCCH type ZFPs are keys to regulating transcriptional activities with the presence of its conserved activator or repressor domains. For instance, rice *OsLIC* and *Ehd4* protein have a conserved EELR domain in its C terminal, which acts as the key component for transcriptional activation of the target genes (Wang et al., 2008). CCCH type ZFPs such as *OsLIC* and *Ehd4* of rice, *AtTZF1* and *AtTZF6* (*PEI1*) of Arabidopsis can bind to the promoter region of target genes (Wang et al., 2008; Bogamuwa and Jang, 2013; Wang et al., 2020). However, in some of the CCCH-type ZFPs, the transcriptional activator domain, called EELR-like, was found to be in N-terminal. This EELR-like domain of ZFPs such as *AtC3H17* and *AtZFP1*were found to play an essential role in transcriptional activation of downstream salt-responsive genes like *AtP5CS1, AtGSTU5, SOS1* and ABA-dependent responsive genes *RD22, COR15A* and *RAB18* (Seok et al., 2016; Seok et al., 2018). In addition to transcriptional activators, some other proteins are transcriptional repressors. For negatively regulating the transcription of target genes, CCCH ZFPs like *GLUB-1-BINDING ZINC FINGER 1 (OsGZF1)* and *ILA1-interacting protein 4 (IIP4)* of rice have repressor motifs. After binding of ZFPs in the promoter region of target genes like *MYB61, CESAs* and *GluB-1*, it negatively regulates the expression of *CESAs* and *MYB61* for secondary wall synthesis and *GluB-1* for accumulation of gluten (Chen et al., 2014; Zhang et al., 2018). In addition to its involvement in transcriptional activities, CCCH ZFPs like *AtTZF1, KHZ1* and *KHZ2* of Arabidopsis also has been reported to be involved in post-transcriptional regulation of gene expression by binding to mRNA through its RNA binding motif (Yan et al., 2017; Pi et al., 2018). It is reported that with the help of the RNA binding domain, ZFPs namely, *OsTZF1* bind to mRNA at the 3’ un-translated region specifically at AU-rich elements (AREs) (Jan et al., 2013). In plants like Arabidopsis, ZFPs like *HUA1* with its RNA binding ability regulates flower development through pre-mRNA processing of *AGAMOUS* (Rodriguezcazorla et al., 2018) and *FRIGIDA-ESSENTIAL 1 (FES1)* promotes the winter annual growth habits in a FRIGIDA-dependent manner by regulating mRNA levels of *FLOWERING LOCUS C (FLC)* (Schmitz et al., 2005). A recent study has found that the splicing efficiency of *FLC* pre-mRNA can be inhibited by *KHZ1* and *KHZ2* (RNA binding ZFPs) and promotes flowering in Arabidopsis through other independent pathways (Yan et al., 2020). Likewise, it has been reported that zinc finger homeodomain proteins (ZF-HD) another class of ZFPs are essential for the induction of flowers in plants like Arabidopsis. As the induction of flowers is affected by environmental stresses, *ZF-HD1* proteins over-expresses during such stress condition and helps in coping with stress through transcriptional activation of genes like *ERD1* (Shalmani et al., 2019).

In plants, through *in vitro* studies, KTEL (V) residue at the N terminus of ZFPs was observed in each zinc finger motif proving to be a key interface for RNA binding (Wang et al., 2008) and plant-specific TZF motif (RR-TZF) & RR sequence in AtTZF1 were found to be the essential motifs for binding to AREs of RNA leading to mRNA degradation (Qu et al., 2014). More recently two putative mRNA binding domains namely RRM and OST-HTH/LOTUS were identified in CCCH type ZFP (*AtC3H18L*) sequence (Xu et al., 2020).

Some ZFPs are co-localized to PBs and SGs. These PBs and SGs have essential roles in the post-transcriptional regulation of several genes, more importantly, during plant tolerance against environmental stresses (Bogamuwa and Jang, 2016). Some ZFPs, namely *AtTZF1, AtTZF4, AtTZF5*, and *AtTZF6* of Arabidopsis, with their cytoplasmic shuttling characteristics, work in association with these PBs and SGs for post-transcriptional regulation of target proteins (Jang, 2016; Seong et al., 2020). Different from the rest of the ZFPs, most of the RING zinc finger proteins have E3 ubiquitin ligase activity and it is involved in post-transcriptional activities of the ubiquitin-proteasome pathway. In this pathway, E3 RING ZFPs help in the recognition of the substrate proteins and degrades or change the activity of target proteins through ubiquitination (Swatek et al., 2019). For instance, *AtATL78, StRFP2*, *AtRZF1, DRIP1, DRIP2, OsDIS1* and *HOS1* post-transcriptionally regulate the target proteins such as *DREB2A, OsNek6, OsSKIPa, AtERF53, ICE1, OsARK4, OsHRK1, AtRma1* and *CaRma1H1*, etc. through ubiquitination (Han et al., 2021) of which many of the target proteins like *CaRma1H1* and *AtRma1* are involved in the transport of the aquaporin PIP2;1 from the ER. It is also reported to have roles in transcriptional and post-transcriptional regulations of different genes involved in ABA-dependent pathways, ROS and Ca^2+^ signalling pathways (Han et al., 2021). Transcriptionally it is involved in the activation of genes encoding enzymes of ABA pathways such as *ABA aldolase, NCED, ZEP* and *short-chain dehydrogenase/reductase* (Vaičiukynė et al., 2019) and post-transcriptionally it is involved in forming a complex with other proteins such as *PP2Cs* and *SnRK2s* for ubiquitination and phosphorylation of other proteins and transcription factors like *protein phosphatase 1* (*CaADIP1*), *CaATBZ1*, *AtAIRP3, bZIP, AtKPNB1, TaSTP, GDU1* and *RD21* which further activates or represses the transcription of ABA-responsive genes (Sah et al., 2016; Joo et al., 2020; Lv et al., 2020; Oh et al., 2020). In the MAPK signalling pathway, zinc finger proteins such as RGLG1 and RGLG2 have been involved in post-transcriptional modification of *MAPKKK18* in response to environmental stress like drought. In ROS and Ca^2+^ signalling pathways, it is involved in transcriptional activation of genes encoding antioxidant enzymes like *SOD* and *POD* in response to environmental stresses (Zang et al., 2016). The ORANGE or OR proteins which are the type of DnaJ type ZFPs with their dual sub-cellular localization abilities, perform transcriptional and post-transcriptional activities in the nucleus and plastids. In the nucleus, it interacts with *eRF1-2* (*eukaryotic release factor 1-2*) for transcriptional regulation of the downstream gene involved in cell elongation of petiole and plastids. It plays a role in post-transcriptional regulation by interacting with *phytoene synthase* to induce biogenesis of chromoplast and accumulation of carotenoid in non-pigmented tissues, simultaneously interacting with *TCP14*, a type of TFs to repress *ELIPs* (*Early Light Induced Proteins*) and biogenesis of chloroplast in de-etiolating cotyledons (Sun et al., 2019; Chen et al., 2021b). Whereas, some reports suggest that DnaJ ZFPs function as molecular chaperones in post-transcriptional or rather post-translational maintenance of structures and functions of its interacting proteins (Ali et al., 2022). The FCS-like zinc finger (FLZ) proteins which are reported to be localized to the cytoplasm and nucleus extensively interact with the kinase subunit of the *SnRK1* complex and act as an adaptor to facilitate the interactions of effector proteins with the *SnRK1* complex. In plants, it was found to be acting as a transcriptional activator by mediating the interactions of *SnRK1* and effector proteins under various environmental stresses, however many of them are reported as negative regulators of *SnRK1* signalling (Jamsheer et al., 2018a and Jamsheer et al., 2018b).

## 3 Role of Zn finger in conferring tolerance to abiotic stress and possible mode of action

**Figure 1:**
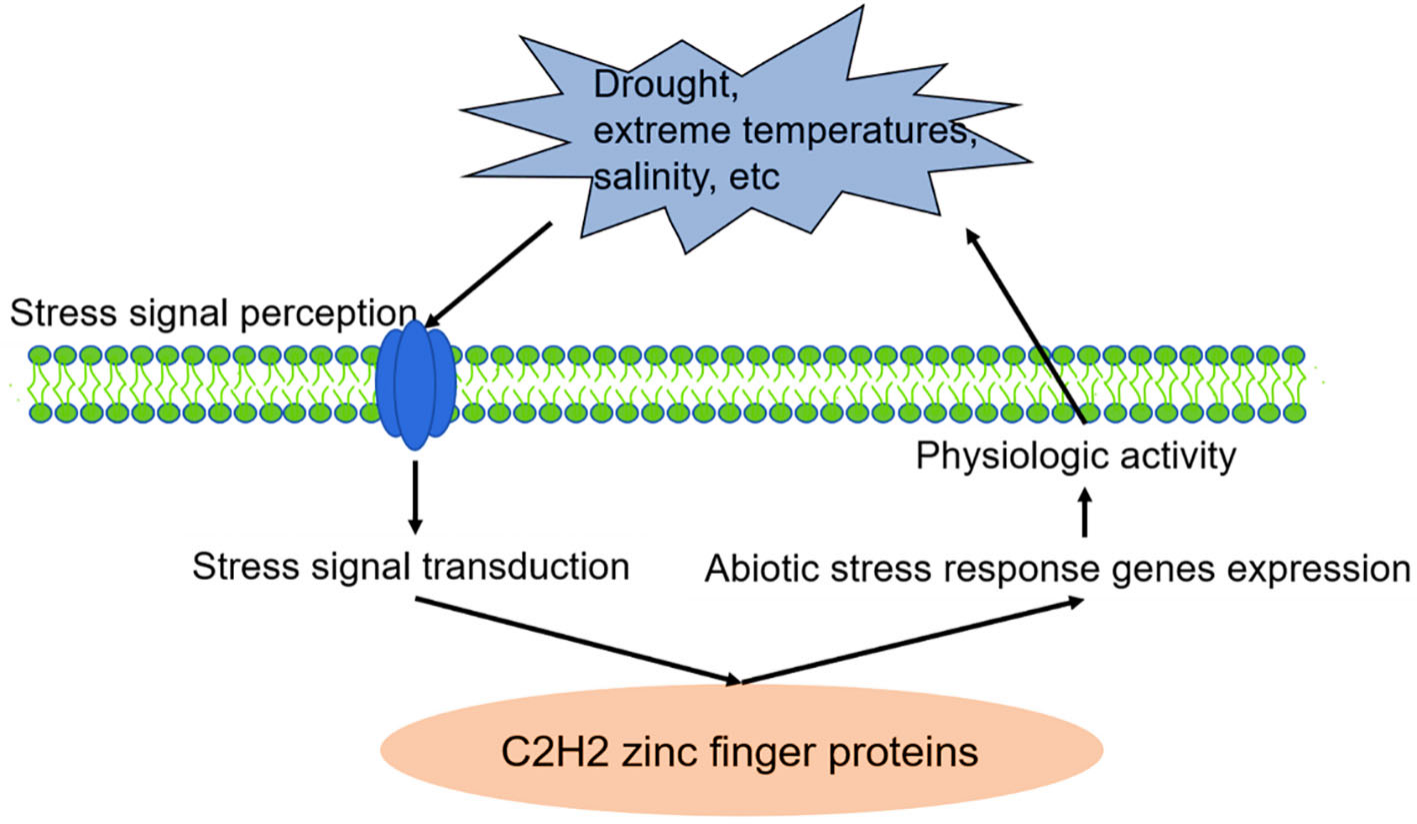
C2H2 zinc finger proteins are involved in plant stress responses. Source: (Liu et al., 2022).

**Figure 2:**
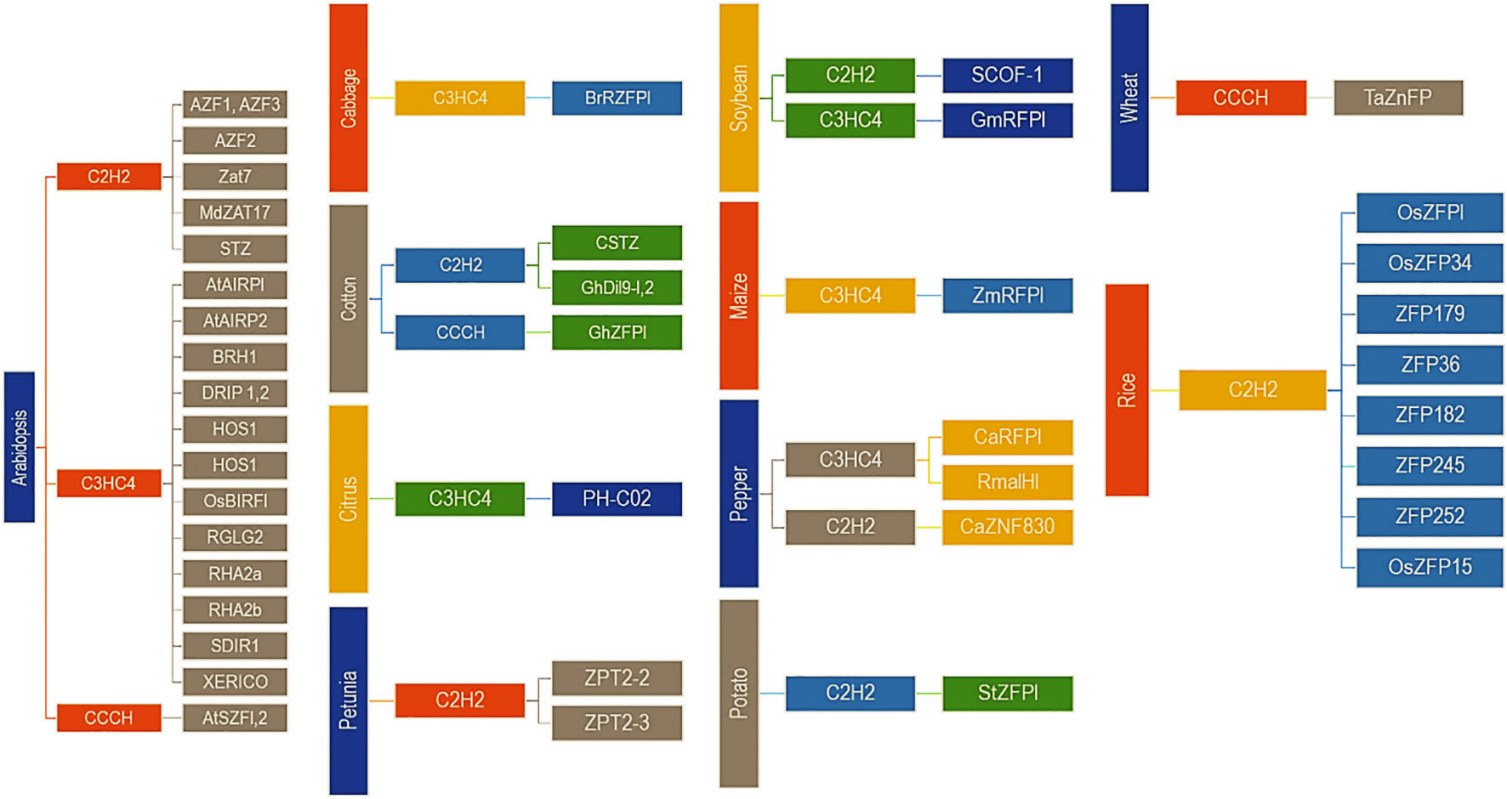
Some important Zinc Finger Proteins (ZNPs) involved in abiotic stress factors of the plants (1^st^ order of the hierarchy is the name of the crop/plant, 2^nd^ order is the type of the protein and 3^rd^ order is the name of the ZNPs) (Source: Modified after Noman et al. (2019) with the permission from the Elsevier Rights Links (https://s100.copyright.com/), Licence No.: 5371200779212, Dated 17^th^ August 2022).

Zinc finger proteins play an extensive role in plant tolerance to various abiotic stress, such as drought, high salt, cold, high light, and osmotic and oxidative stresses (Wang et al., 2019; Han et al., 2020). In the process of external stress resistance, plants have evolved a set of complex and effective defence mechanisms, including signal perception, signal transduction, transcriptional regulation and response, to reduce or avoid damage to plants and ensure their average growth (**Figures 1**, **2**) (Noman et al., 2019; Liu et al., 2022).

Several studies found that Zn-finger motifs significantly function during abiotic stress response in plants. In the first report, a wide range of Zn-binding motifs has been recognized and termed as Zn-fingers. Since the zinc finger motifs regulate the function of stress-adaptation genes. The Zn-finger was first reported as a repeated Zn-binding motif, comprising conserved cysteine (Cys) and histidine (His) ligands, in *Xenopus laevis* oocytes as a transcription factor (TF) IIIA (TFIIIA) (Laity et al., 2001; Ciftci-Yilmaz and Mittler, 2008; Kielbowicz-Matuk, 2012; Lyu and Cao, 2018). In the proteins where Zn^2+^ is mainly attached to amino acid residues and thus espousing a tetrahedral coordination geometry. The physical nature of Zn-proteins, defining the attraction of Zn-proteins for Zn^2+^, is crucial for having an in-depth knowledge of how a Zn^2+^ facilitates their characteristic function and how proteins control its mobility (intra and intercellular) as well as cellular availability.

**Figure 3:**
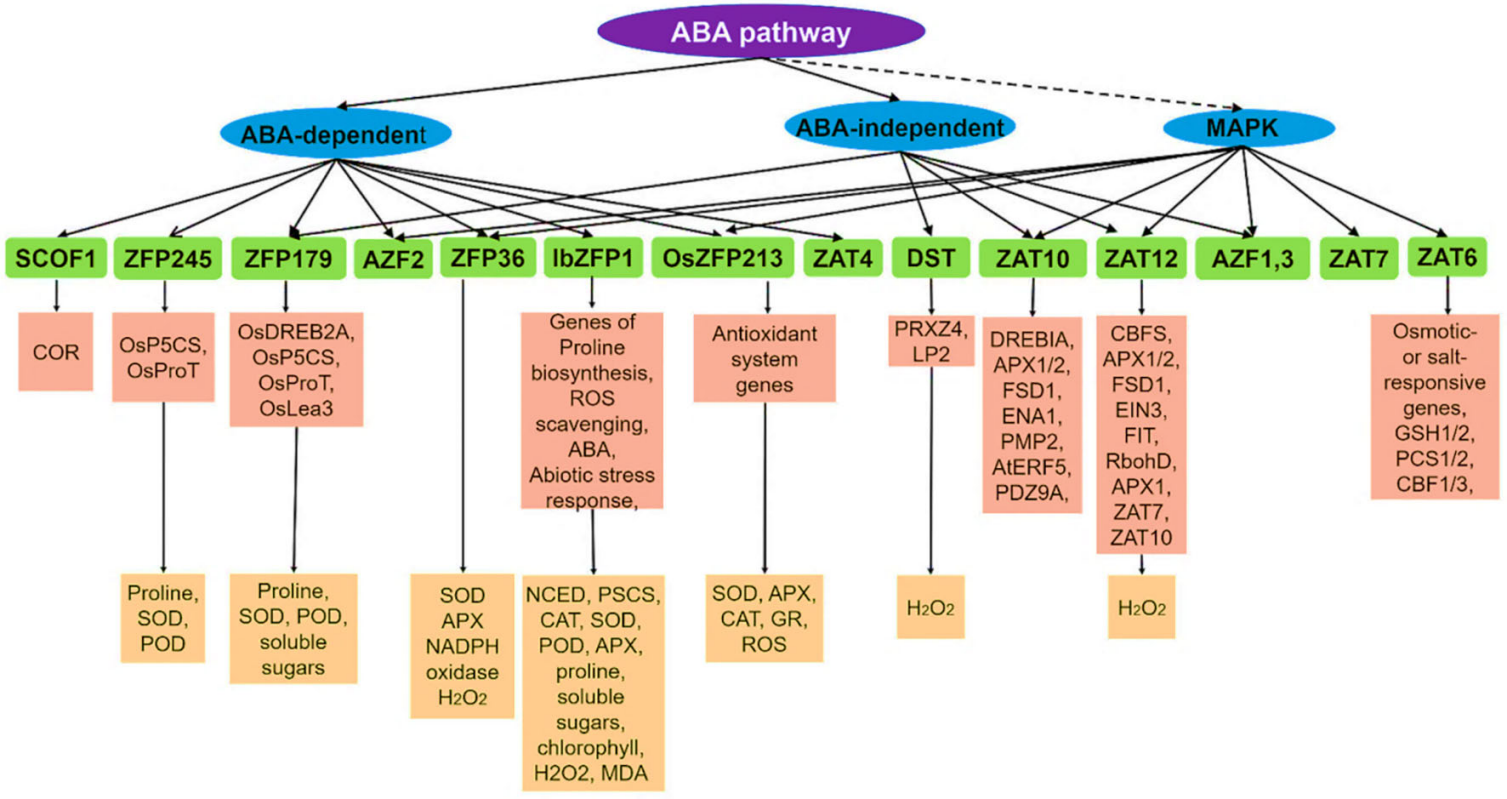
The signalling pathways of zinc finger proteins during abiotic stress response in plants. Note: The lines marked as solid indicate regulation, and the dashed lines indicate putatively. The C2H2 zinc finger proteins are SCOF-1, ZFP245, ZFP179, AZF1/2/3, ZFP36, IbZFP1, OsZFP213, ZAT4, DST, ZAT10/STZ, ZAT12, ZAT7 and ZAT6. Source: (Liu et al., 2022).

Many C_2_H_2_-type zinc finger proteins involved in the abiotic stress signalling pathway were identified based on stress induction, mutant, compliment, and ectopic expression analysis. Phytohormones are responsible for abiotic stress resistance and participate in the process of response to various stresses *via* C_2_H_2_-type zinc finger proteins, especially ABA (Abscisic acid) (Roychoudhury et al., 2013; Ku et al., 2018; Takahashi et al., 2018; Chong et al., 2020). ABA, acting as a pivotal regulator of abiotic stress responses in plants, induces the expression of stress-related genes and triggers a range of adaptive physiological responses under abiotic stress conditions in the plant (Ku et al., 2018; Takahashi et al., 2018; Chong et al., 2020). C_2_H_2_ zinc finger proteins regulate plants in response to abiotic stresses through two ABA-mediated signal pathways: ABA-dependent and ABA-independent signal pathways (Smekalova et al., 2014; Ku et al., 2018; Takahashi et al., 2018; Chong et al., 2020). In addition to the ABA signal pathway, C2H2 zinc finger proteins enhance abiotic stress resistance by the MAPK (mitogen-activated protein kinase) signaling pathway (Smekalova et al., 2014; Ku et al., 2018; Takahashi et al., 2018; Chong et al., 2020; He et al., 2020; Lin et al., 2021). MAPKs, as highly conserved signaling transduction modules, play an essential role in regulating responses to adverse environmental stresses (Lin et al., 2021). Typically, a MAPK module is composed of at least three protein kinases, including MAPK (MPK), MAPK kinase (MAPKK/MAP2K/MKK/MEK) and MAPK kinase (MAPKKK/MAP3K/MEKK). The MAPK cascade amplifies and conveys stress signals from signaling receptors to downstream stress response factors through a sequential phosphorylation manner (De Zelicourt et al., 2016; He et al., 2020; Lin et al., 2021). Thus, C_2_H_2_ zinc finger proteins regulate abiotic stress responses *via* both the ABA signaling pathway and MAPK signaling transduction pathway and constitute a certain degree of crosstalk and a complex regulatory network (**Figure 3**) (Liu et al., 2022).

### 3.1 Salinity

In many regions of the globe, soil salinity is significant abiotic stress that inhibits plant production. Salinity stress creates nutrient imbalances, is a source of harmful ions, and alters the osmotic condition of plants (Yaghoubian et al., 2021). It has been reported that more than 108 × 108 km^2^ of land throughout the world are affected by salinity (Riaz et al., 2019). Due to salinity and problematic soils, millions of hectares in the humid areas of South and Southeast Asia that are technically suitable for various field crops, particularly rice cultivation, are left uncultivated or are cultivated with extremely poor yields. (Mainuddin et al., 2019; Mainuddin et al., 2020). In addition, mineral shortages and toxicity often exacerbate the issue of salinity since it seldom occurs alone. These soil stresses change in magnitude and interactions throughout time and space, making long-term adaptation dependent on its degree of tolerance to all environmental stresses (Sarkar et al., 2022). Moreover, the ill response of soil and water salinity in the different crops varied significantly.

As transcription factors, zinc finger proteins (ZNPs) play an essential part in a wide variety of cellular processes, including RNA binding, transcription control, stress tolerance, and plant growth and development in response to phytohormones) (Noman et al., 2019). Scientists have identified a large number of zinc finger proteins in higher plants that can regulate the various environmental cues (Yu et al., 2015). Around 189 stress-induced zinc finger proteins especially for indica rice and maize have been classified and amongst them, Cys2/His2- and CCCH-types have received greater attention (Agarwal et al., 2007) Multiple C2H2-type zinc finger proteins in rice, including ZFP36, ZFP179, ZFP182, ZFP245, and ZFP252, have been implicated in salt, drought, and oxidative stress responses (Xu et al., 2008; Wang et al., 2015; Wang et al., 2022a). It has been found that drought and salt tolerance in rice are improved by overexpressing the ZFP252 zinc finger protein gene by elevated synthesis of free proline and soluble sugars (Xu et al., 2008) and increased ROS scavenging activity (Huang et al., 2009). Huang et al. (2007) found that the production of ZFP182 in transgenic tobacco or overexpression in rice plants boosted their salt tolerance, ZFP182 may have a curative function in salt tolerance in plants. Wang et al. (2022a) reported that some C_2_H_2_ type Zinc finger protein plays a significant role in salinity tolerance in rice seedlings by enhancing ABA catabolism. They also stated that overexpression of OsZFP15-like Zinc finger protein increased the production of reactive oxygen species (ROS) and decreased tolerance to oxidative stress, resulting in increased salinity stress tolerance in rice seedlings. The A20/AN1-type (ZmZnF1) and ring-binding type (ZmZnF2) zinc finger proteins found in maize kernels are reported to induce by ABA, mannitol and NaCl stresses. Similarly, simultaneous overexpression of these zinc finger proteins in transgenic lines of rice significantly increased the Na induced stress tolerance (Yu et al., 2015). It has been observed that transgenic Arabidopsis plants that constitutively produce the Cys2/His2 zinc finger protein Zat7 exhibit slower growth and development and a higher degree of tolerance for the effects of salt stress and the ability to tolerate high salinity is lost when the EAR motif of Zat7 has a mutation or deletion (Ciftci-Yilmaz et al., 2007). Wang et al. (2022b) found that the overexpression of C_2_H_2_-type zinc finger protein ‘MdZAT17’ in transgenic apple and Arabidopsis reduces the sensitivity to abscisic acid (ABA) and regulates salt tolerance positively. They also reported that the growth of both wild-type and transgenic *Arabidopsis* seedlings was inhibited under salt stress, but the growth shyness of transgenic plants was pointedly lower than that of wild-type seedlings. In another study (Xu et al., 2022) has been identified 36 B-box (BBX) family of proteins in maize consists of zinc-finger transcription factors that have a significant role in the regulation of different abiotic stresses, including drought and salinity.

### 3.2 Soil moisture

Water is one of the fundamental inputs for the growth and yield of any arable crop. Still, both excess and deficit water supply in different growth stages can significantly reduce crop production. In the present era of climate change, aberrant weather conditions have increased the excess and deficit supply of soil moisture in most growing regions worldwide. Crop physiological and biological adaptability with the excess and deficiency supply of soil moisture has emerged as a potential research question (Osakabe et al., 2014). Multiple Zinc finger proteins have been identified from various crops has a definite role concerning drought and excess moisture stress of the field crops. Numbers of ZFPs have been identified in transgenic rice that has significant functions in enhancing drought, and excess moisture tolerance (Huang et al., 2009; Li et al., 2013; Wang et al., 2022a). Huang et al. (2009) reported that ZFP245 type ZNPs have significantly enhanced the cold and drought tolerance in rice by augmenting free proline and antioxidant concentration in transgenic rice plants. They further suggested that ZFP245 transgenic rice plants under 14 days drought stressed condition has survived at 70-80% with 7 days recovery period. It has been postulated that under cold or drought stress, ZFPs boost the SOD and POD enzymatic activities in transgenic rice seedlings and thereby help to enhance the abiotic stress tolerance by triggering the ROS-scavenging mechanism (Rizhsky et al., 2004). In harmony to the previous findings, (Sakamoto et al., 2004) found that two Cys-2/His-2-type ZNPs viz. AZP2 and STZ in transgenic Arabidopsis have significantly overexpressed under drought-stressed conditions and facilitated the plant to tolerate drought stress. (Gao et al., 2012) reported that ‘CgZFP1’, a Cys2/His2 type ZFP isolated from Chrysanthemum has a significant role in regulating drought and salinity stress in transgenic Arabidopsis. In severer drought conditions, abscisic acid plays an important role in enhancing the plant’s leaf senescence rate, reducing the yield. It has been found that a Cys-2/His-2-type ZNP, ´MdZAT10´, reduced the sensitivity to abscisic acid in apples and in addition to that, MdZAT10 (overexpressed in *Arabidopsis*) has a beneficial effect on seed germination and seedling growth (Yang et al., 2021). In another study, Mukhopadhyay et al. (2004) isolated a ZNP from rice viz. “OSISAP1” (induced by abscisic acid) overexpressed in transgenic tobacco increased tolerance to cold, dehydration, and salt stress at early growing stages. Giri et al. (2011) reported that Stress-associated ZNP isolated from rice viz. A20-AN1 increased the abiotic stress tolerance in transgenic *Arabidopsis* plants.

Most of the study suggested that ZNPs isolated from various plant sources involved in regulating abiotic stress factors has manually controlled the drought and salinity stress simultaneously (Huang et al., 2009; Lawrence et al., 2022). But application or overexpression of ZNPs in different transgenic crops is precise in nature.

### 3.3 Temperature

**Figure 4:**
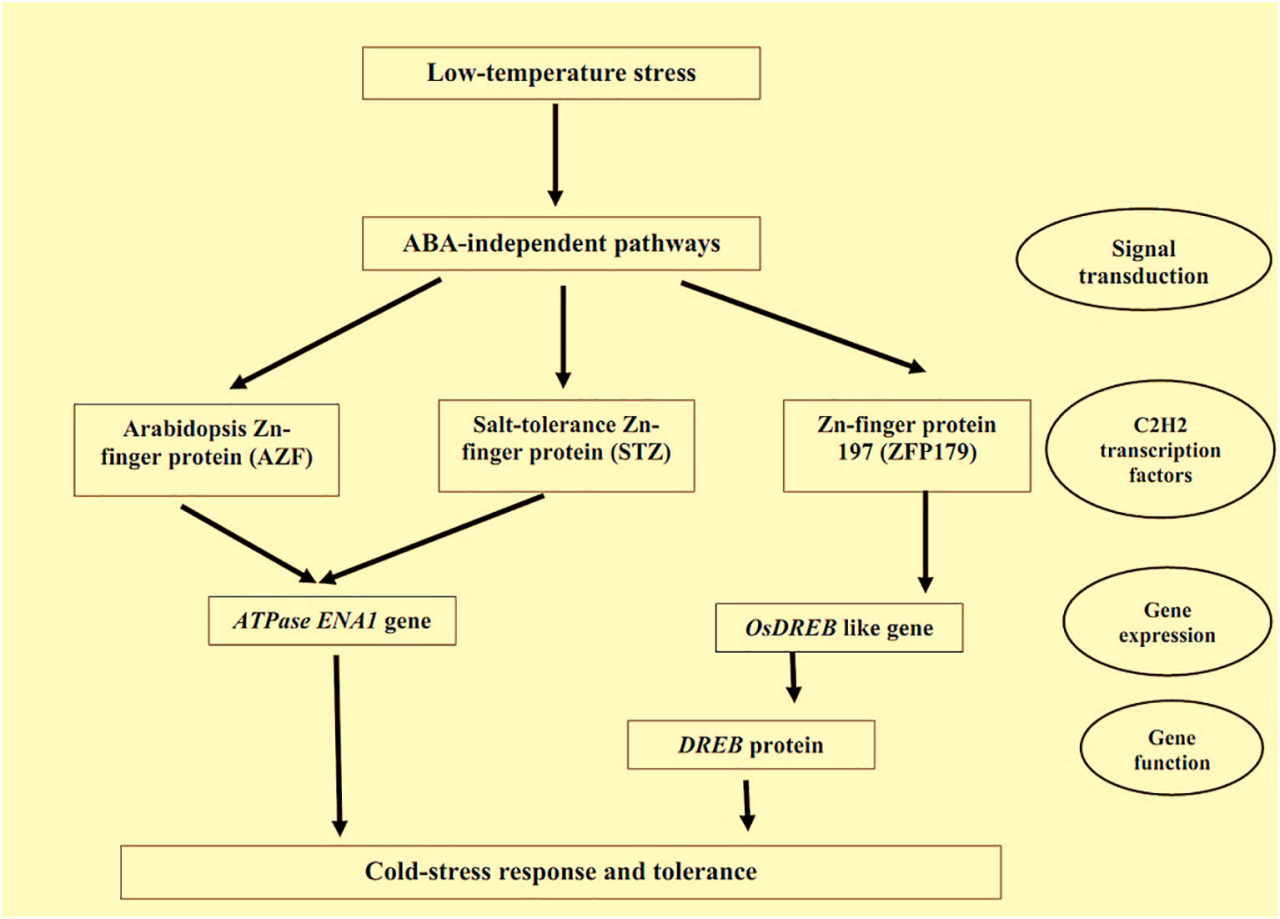
ABA-independent pathways for cold-tolerance by C2H2 Zn-finger proteins. Note: Zn-finger proteins also regulate the several pathways of plants under heat-induced stresses. Huang et al. (2008) found A20/AN1-type Zn-finger proteins in japonica rice regulating the heat-induced stresses in the plants. Moreover, they reported that Zn-finger protein, ZFP177 was responsive to heat stress tolerance in plants. Overexpression of Zn-finger protein, ZFP177, is also responsible for the heat tolerance in tobacco plants (Mukhopadhyay et al., 2004).

Temperature stress due to both cold and high temperatures is considered a vital abiotic stress a plant faces during its growth and development (Liu et al., 2018). Thus, a clear-cut concept about the bio-physical-chemical impacts of temperature on a plant and its subsequent response mechanism is crucial to breeding improved stress-tolerant cultivars (Liu et al., 2018). Many Zn-finger proteins are responsible for mitigating the different temperature-related stresses in plants. CCCH Zn-finger proteins can control the expression of cold-induced genes; these proteins can improve cold tolerance in plants. Lin et al. (2011) found that the expressions of the cold-temperature genes viz. COR15A, RD29A, KIN1 etc. were upregulated in the cold-stress tolerant different Arabidopsis lines. Enhancement of the cold-stress tolerance in the plants can also be explained through signalling pathways of ABA, which are controlled by CCCH Zn-finger proteins (Xie et al., 2019). Transgenic switchgrass lines with induced Zn-finger protein, PvC3H72, survived even at –5°C temperature, while the lines with the low Zn-finger proteins could not survive (Xie et al., 2019). Zn-finger protein, DgC3H1, can improve the proline and soluble sugar levels in *Chrysanthemum* plants along with increased-level of SOD and POD, which ultimately make the plants able to survive under cold stress (Bai et al., 2021). C_2_H_2_ Zn-finger proteins also play a pivotal part in mitigating cold stress by regulating the ABA pathways. Kim et al. (2001) reported that the overexpression of C_2_H_2_ Zn-finger proteins results in cold-stress tolerance in soybean and tobacco by controlling ABA-response elements which facilitate COR gene-expression responsible for developing the plant cold-tolerance. The ABA-independent pathway for cold tolerance through C2H2 Zn-finger proteins is represented in **Figure 4**. In addition to the above two Zn-finger proteins, the AZF: Arabidopsis Zn-finger protein and STZ: salt-tolerance Zn-finger proteins are also responsible for cold-stress tolerance in Arabidopsis (Kodaira et al., 2011). The AZF genes, AZF, AZF2, AZF3, and STZ gene regulate the ABA-dependant pathway of Arabidopsis by regulating the ATPase gene, Na+, and Li+ outflows in plants (Lippuner et al., 1996).

### 3.4 Heavy metal (HM)

#### 3.4.1 HM-tolerance through cellular layer modification

For any HM stress, the plant has a three-tier strategy including absorption or isolation of HM inside the plant, HM removal through a series of chelating mechanisms and ROS removal which are accumulated during HM stress. Other than this a few subsidiary events of the HM-tolerance mechanism include an impedance of HM transport within the plant through HM binding to the cytoderm (composed of cellulosic material) (Chen et al., 2019). Few HM ions bind to the active groups (-COOH, -OH) of cellulose (inside cytoderm) which reduces the quantity of HM that enters the protoplasm resulting in damage alleviation caused by HM (Nedelkoska and Doran, 2000; Clemens et al., 2013). Three types of zinc finger transcription factors (ZF-TFs) (GATA-type, CCCH-type, and C2H2-type) are expressed differentially and reported to be up-regulated under cadmium stress in cotton roots, some of which are shown to be associated with secondary cell wall biosynthesis (Chen et al., 2019). Up-regulation in the homologs of cellulose synthase genes during cadmium stress reveals the involvement of ZF-TF in cellular layer modification as one of the possible modes of action for imparting HM-stress tolerance in cotton plants.

In *Arabidopsis thaliana*, upon exposure to HMs exhibited some interesting findings. With the help of yeast-two-hybrid model when interaction among the different members of the HIPP family and the related zinc finger TF or transcription factors, have borne a particular interaction pattern of ATHB29 and HIPP proteins (of cluster III). Thus, a purposeful connection among ATHB29 and HIPP26 is also shown by experiments conducted with HIPP26 (mutants), displayed reformed expression pattern levels of such genes earlier known to be controlled by ATHB29 (Barth et al., 2009). Chakrabarty et al. (2009) while investigating genome‐wide expression pattern of Zn-finger proteins in plants exposed to As^3+^ and As^5+^ reported a contrasting scenario. Proteins of the zinc‐finger family were found to be downregulated under As^5+^ stressed situation whereas, Zinc‐finger C3HC4‐type proteins were found to exhibited both up and down regulated expression profile upon exposed to As^3+^ stressed situation. Authors like Abercrombie et al. (2008); Chakrabarty et al. (2009); Tripathi et al. (2012) have presented the expression profile of *Oryza sativa* (monocot) and *Arabidopsis thaliana* (dicot). Under As stressed condition both exhibited a downregulated Zn-finger protein expression profile. Shah et al. (2022) observed that spermine (Spm), a polyamine compound upon supplanting in common bean (*P. vulgaris*) exposed to As stress have enhanced the expression of PvC3H24, PvC3H25, PvC3H26 and PvC3H27 (Zn-finger proteins). The authors concluded that, Spm confer tolerance to As induced phyto-toxicity by modulating polyamine metabolism, antioxidant defense system along with facilitating (as enhancer) for zinc-finger proteins related genes expressions. Huang et al. (2012) reported some interesting findings while analysing the transcriptomic profile of rice roots exposed to As stress. among the 231 TFs, the zinc-finger protein (expressed particularly in inflorescence meristem or ZIM) had a share of only 3.46%. The authors have concluded that under As stress ZIM-TF were enhanced in a noteworthy fashion.

#### 3.4.2 HM-tolerance through HM-homeostasis strategies

HM homeostasis strategies mainly involve the phytochelatin, metallothionein and metallochaperones (responsible for safe transport of metal ions inside the cell) induction (Harrison et al., 2000; de Abreu-Neto et al., 2013; Zhang et al., 2014) as well as vacuolar sequestration mediated by phytochelatin binding (Yang and Chuand Chu, 2011). Barth et al. (2009) found a functional association between a ZF-homeodomain (ZF-HD) TF (known as ATHB29) and arrays of HIPP26 (a type of metallochaperone protein) in *A. thaliana* under HM stress by double checking mutant line assay (mutated for HIPP26 functional loss) for the expression of stress-responsive genes which showed that the genes up-regulated under the influence of ATHB29 are inhibited in the absence of functional interaction between HIPP26 and ZF-HD.

#### 3.4.3 HM-tolerance through protein-protein interactions (PPIs) and signal transduction

Plant stress-associated proteins (SAPs) contain ZF domains (either [Cys2-Cys2]_n_ finger motifs or [Cys and His]_n_ residues; ‘n’ represents multiple numbers of motif repeat) at the N- or C-terminal (Jin et al., 2007). These SAPs can be a key player in stress signaling through protein-protein interactions (PPIs) *via* their ZF-domain (Opipari et al., 1990; Kanneganti and Gupta, 2008). Additionally, SAP10 of A. thaliana was found to be coding for a nuclear or cytoplasmic protein that might act early in the signal transduction of the HM-stress tolerance response (Ströher et al., 2009; Dixit and Dhankher, 2011). Many of the HM defense responses shown by the plants are due to the major contribution of cellular receptors involved in signaling cascades like MAPK (three-tier phosphorylation module) (Rodriguez et al., 2010; Sinha et al., 2011; Jalmi and Sinha, 2015) which modulates downstream WRKY and ZAT TFs (containing ZF motifs) (Ogawa et al., 2009; Opdenakker et al., 2012). PPI (protein-protein interaction) study performed by Nguyen et al. (2012) between ZAT-TF (ZAT10; a ZF-containing TF) and Arabidopsis MAPK (MPK3, MPK6) under HM stress has paved a conceptual understanding that MAPKs are involved in HM stress signaling either through ZF-TFs or transcription factors containing ZF-motifs. Major plant stress tolerance responsive signaling factors includes EF-Tu receptor, ETR1/ETR2, SIT1, ER etc. Ca signaling is important in heavy metal stress hormone signaling, as is MAPK signaling, which uses Calmodulin, a Calcineurin B-like protein, and Ca dependent kinase (Steinhorst and Kudla, 2014). Among these, MAPK signalling is one of the major signalling pathways involved in alleviating heavy metal stress. Two transcription factors, viz., MPK3 and MPK6, get activated under high Cd stress mediated by ROS signalling (Liu et al., 2010). Calcium- and cadmium-responsive mitogen activated protein kinase (MAPKKK) in Arabidopsis remains a major signal transduction protein component (Suzuki et al., 2001).

#### 3.4.4 HM-tolerance through ROS-detoxification

HM stress always generates oxidative stress and causes destabilization in the balance between ROS and the antioxidant system (Zhang et al., 2013; Jin et al., 2016; Yin et al., 2016; Xu et al., 2019). Different transcription factors (TFs) families including zinc finger-TFs are involved in such ROS-mediated stress responses (Singh et al., 2019). HM accumulation ROS mediated functional disruption of biomolecules among several other damages (Stohs and Bagchi, 1995; Cuypers et al., 2009; Haider et al., 2021). Under HM-stress SlRING1-ZFP overexpression in tomatoes led to more chlorophyll content and photosynthetic rate. Moreover, the maximal photochemical efficiency of photosystem II was evidently improved by SlRING1-ZFP mediated minimization of ROS levels and electrolyte leakage (Ahammed et al., 2020). Soybean ZFP (GmRZFP1) and Arabidopsis ZFP (AtOHRP) are reported to be involved in oxidative stress (activity induced by ROS) in plants thereby promoting ROS scavenging enzyme systems (Wu et al., 2010; Li et al., 2015). Putting together, zinc finger protein (ZFP) is involved in HM stress alleviation and detoxification of ROS as two underlined strategies of HM tolerance. Abscisic acid enhances plant stress response mechanisms under drought stress. Ring-zinc finger protein overexpression enhances genes like AtNECD3, which codes for a key enzyme in ABA biosynthesis (Ko et al., 2006). Zinc finger protein also enhances major genes’ expression (ABA3, ABI5, etc.) responsible for ABA biosynthesis in rice (Zeng et al., 2015). Zinc finger protein ZFP-185 regulates ABA and GA synthesis modulation, which enhances stress response (Zhang et al., 2016). Flowering and gibberellin biosynthesis are also controlled by ZFP in *Chrysanthemum* (Yang et al., 2014). The effect of different Zinc finger protein in mitigating various abiotic stresses has been presented in **Table 1**.

**Table 1:** The effect of different Zinc finger protein in mitigating various abiotic stresses.

Stress	Crops	Mechanism involved	Effect	Reference
Heavy Metal	*Arabidopsis thaliana*	MAPKKK	Alterations in Auxin Homeostasis	Kovtun et al. (2000)
	*Arabidopsis thaliana*	PIN	Auxin transport	Tognetti et al. (2010)
	*Oryza sativa*	MAPK	Decreased ROS induced root cell death	Tsai and Huang (2006)
	*Oryza sativa*	GSH and Sodium nitropruside	By product S nitrosoglutathione generates bioactive NO	Mostofa et al. (2015)
	*Medicago sativa*	MAPKs	Cellular Signal induction	Jonak et al. (2004)
	*Gossypium hirsutum*	Reduced Glutathione (GSH)	Multivesicular body formation induced, structural integrity of cellular components	Khan et al. (2020)
Drought	*Oryza sativa*	OsC3H47, OsTZF1, OsTZF2	Involves in ABA and JA Production modulation	Han et al. (2021)
	*Oryza sativa*	OsC3H10	Enhances expression of LEAs, GLPs and PR protein	Seong et al. (2020)
	*Glycine soja*	GsZFP, a new cis2/His2 type Zinc finger protein	Plant development and stress response	Luo et al. (2012)
	*Arabidopsis thaliana*	AetTZF1	Enhances expression of CBF1, CBF2, DREB2A, COR47	Jiang et al. (2014)
	*Malus domestica*	MdZAT10	Negative regulator of Drought resistance	Yang et al. (2021)
	*Malus domestica*	MdDof54	Development and stress response	Chen et al. (2020)
Salinity	*Festuca arundinacea*	FaZNF	Regulation of Salt stress response pathway	Martin et al. (2012)
	*Arabidopsis thaliana*	ZAt7	Senescence and defense	Ciftci-Yilmaz et al. (2007)
	*Chrysanthemum sp*	CgZFP1	Osmotic adjustment, ROS scavenging, and ion homeostasis	Gao et al. (2012)
	*Arabidopsis thaliana*	ZPT2	Transcriptional repressor	Sakamoto et al. (2004)
	*Ipomea batatus*	IbZFP	Growth, developemnt and homeostasis	Wang et al. (2016)
Cold	*Petunia hybrida*	PhZFP1	Modulation of galactinol synthase	Zhang et al. (2022)
	*Gossipium hirsutum*	GhZAT	Transcription regulator	Fan et al. (2021)
	*Panicum virgatum*	PvC3H72	Transcriptional Activator factor	Xie et al. (2019)
	*Arabidopsis thaliana*	GmZF1	Modulates cold responsive genes	Yu et al. (2014)
	*Nicotiana tabacum*	OSISAP1	Stress response inducer	Mukhopadhyay et al. (2004)

#### 3.4.5 HM-tolerance through ubiquitin proteosome-mediated degradation of misfolded or altered protein

Under abiotic stress, including HM, many functional proteins undergo the ubiquitin proteosome-mediated degradation process due to the altered quaternary structure of the protein (resulting in misfolded design) and hence no longer required for average growth and development (Blasiak et al., 2019). This way of aberrant protein identification and removal ensures better survival of plants under stress conditions (Stone, 2019). Most RING zinc finger proteins have E3 ubiquitin ligase activity (Joazeiro and Weissman, 2000; Ahammed et al., 2020) similar to the last enzyme of the ubiquitin-proteasome pathway (which plays a specific role in recognising target substrates and then degrades the target protein or changes the activity of the target protein). Hence RING-ZFP can be engaged in ubiquitin proteosome-mediated degradation of the misfolded or altered protein (Swatek et al., 2019) as a stress tolerance mechanism.

#### 3.4.6 HM-tolerance through domain interaction with metal ions

Further bioinformatics analysis on RING-ZFP (possess intra- and extra-cellular domains) has already revealed research findings on extracellular domain binding (negatively charged) of RING-ZFP to the positively charged extracellular harmful metal ions suggesting a sensor-like activity of ZFP upon HM accumulation. Additionally, the intracellular domain of RING-ZFP interacts with and promotes ROS-mediated ABA signalling (Suh et al., 2016).

#### 3.4.7 HM-tolerance through GSH-dependent pathway and chelation-based vacuolar sequestration

A GSH-dependent pathway and phytochelatin (polymerized GSH encoded by PCS)-conjugated vacuolar sequestration are two established mechanisms contributing to HM-tolerance (Flores-Cáceres et al., 2015; Hernández et al., 2015; Jozefczak et al., 2015). Cd-tolerant Arabidopsis phenotypes resulted from ZAT6 over-expression, when undergoing BSO (an irreversible inhibitor of GSH biosynthesis; γ-glutamylcysteine synthetase; Reliene and Schiestl (2006) treatment, completely loses its HM-tolerance ability which implies that the ZAT6-mediated enhanced Cd tolerance is GSH-dependent (Chen et al., 2016). Furthermore, Chen and his co-workers have also found GSH1 (encoding γ-glutamylcysteine synthetase) as the transcriptional target of ZAT6 through qRT-PCR (outcome: positive regulation of GSH1 by ZAT6) and transient expression analysis (outcome: activation of GSH1 promoter activity by ZAT6). Putting together, it is clear that GSH1 which regulates HM tolerance in Arabidopsis is under transcriptional control of ZAT6, a ZF-TF. Phytochelatin synthesis was found to be under ZAT6 (acts as TF for gene encoding PCS1 and PCS2) transcriptional regulation too under HM-stress (Chen et al., 2016). Phytochelatin synthesis additionally augments the effect of GSH towards Cd-tolerance by accumulating Cd followed by phytochelatin-conjugated vacuolar sequestration (Wawrzynski et al., 2006; Lin and Aarts, 2012). Saad et al. (2018) demonstrated that LmSAP overexpression in tobacco seedlings enhanced the expression of several genes encoding metallothionein proteins (thiol group of cysteine amino acid residue binds with metal ions and/or ROS, (Yuan et al., 2008; Hassinen et al., 2009).

#### 3.4.8 HM-tolerance findings through QTL analysis

Out of twenty-three sequences of sorghum gene homologs identified by BLASTP searches of the Xtxp270-*QTL* genomic region on chromosome 10, two gene homologs encoding ZFPs (SbZFP17 and SbZFP346) were found to be up-regulated under HM stress (Abou-Elwafa et al., 2019). Another HM-tolerance study on Arabidopsis reported decreased HM-tolerance upon mutation in the gene encoding a ZFP (ZAT6) (Chen et al., 2016).

### 3.5 Radiation/high light stress

Over-expression of a gene encoding an HM (Si) transporter in rice was found to promote strong cell membrane structure and activate regulators (ZF protein *viz*. Lsd1 and dof, protein kinase domain) of the UV-B tolerance signal transduction pathway (Fang et al., 2011), suggesting the possible involvement of zinc finger motif in irradiance-induced signalling.

The B‐box (BBX) proteins are a family of zinc finger TFs containing one or two BBX motifs, which has already elucidated its role in PPI (Gangappa and Botto, 2014; Zhang et al., 2017). Fang et al. (2019) monitored the transcript levels of eight BBX genes in the apple, which were significantly induced by UV‐B radiation. Further, MdBBX20 overexpression in apple callus promoted the expression of structural genes encoding anthocyanin pigments and their subsequent accumulation under UV‐B radiation, possibly by its transcriptional coactivator role (promoter modulation of several proteins) that coordinates with MdHY5 (Fang et al., 2019). In fact, the direct relationship between the expression profile of rhl41 (encode for a ZFP) and collective accumulation of anthocyanin and chlorophyll under UV irradiation suggests the disguised role of zinc finger in photo-protection and increased level of photosynthetic efficiency (both are evolved as strategies of light and radiation tolerance in plants) respectively (Iida et al., 2000).

Exposure of plants to light intensities that exceed the electron utilization capacity of the chloroplastic photosystem (PS) and light-harvesting complex (LHC) dramatically impacts nuclear gene expression. In this regard, the interaction and genetically association (binding component unknown to date) between GATA-type zinc finger-TF (ZML) and CryR1 (cryptochrome involved in growth and regulation) induce the formation of ZML heterodimer regulating the expression of photo-protective genes (Shaikhali et al., 2012).

Protective role (as a shield of LHC of PS-I, II and as an antioxidant; quenching of excited electrons during an imbalanced state of excitation transfer at the LHC) of flavonoid (anthocyanin) class of metabolites are very prominent under light and irradiance stress (Hughes et al., 2014; Tattini et al., 2014). HY5 (a type of Leucine zipper TF) positively regulates light‐responsive gene expression (Nawkar et al., 2017) through interaction (*via* phytochrome interacting factor) with BBX-ZFP resulting in anthocyanin accumulation under light and/or radiation stress (Wei et al., 2016; Zhang et al., 2017). MdBBX20 responds to UV‐B signaling and forms an active heterodimer with HY5 (one of the key regulatory factors for UV-B response; facilitating the transcriptional activity of HY5 (An et al., 2019; Fang et al., 2019).

Modulation in antenna size of LHC is another approach adopted by the plant to tolerate incoming high light and radiation stress (Rossel et al., 2002; Kimura et al., 2003; Mittler et al., 2004). Genetic alteration of ZAT10 (ZF-TF) has a modulatory impact on high light-induced transcriptome (Rossel et al., 2002) whose products are targeted to chloroplast suggesting the involvement of ZF-TF (zinc finger motif) in plants’ manipulation of chloroplastic apparatus.

### 3.6 Elevated CO_2_ stress

The experiment setup (95% CO_2_+1% O_2_ treatment; Artificial high-CO_2_ atmosphere) designed by Jamil et al. (2019) reported abundant levels of C_2_H_2_-type ZFP (DkZF1-5) transcripts. Even though there are no direct *in-vivo* reports on ZF-motif-mediated stress tolerance under the influence of elevated CO_2_ to date but still, the existing elucidated protein-protein interactions among the expressed product of DkZF1-5 transcripts might have some synergistic regulatory role under CO_2_ stress. Notably, comparative transcriptome analysis had already unveiled up-regulation in stress-related TFs (WRKY; domain containing C2HC ZF motif) (Rushton et al., 2010) in immature California grapes under CO_2_ gaseous treatment (Rosales et al., 2016; Romero et al., 2019). ABA phytohormone maintenance (balance between anabolism and catabolism) and their optimal levels are influenced by elevated CO_2_ (linear relationship between CO_2_ concentration and ABA synthesis) (Seo et al., 2000; Iuchi et al., 2001; Xiong et al., 2001; Xiong et al., 2002; Xiong and Zhu, 2003; Zou et al., 2007). Both of the above concepts indicate the possible involvement of WRKY with ABA-inducible and -repressible genes (under the influence of HVA22 promoter; Zou et al. (2004) under elevated CO_2_ suggesting ABA-mediated stomatal response to CO_2_ stress in plants. The present interpretation and previously made elucidations suggest the involvement of WRKY-TF as a functional node integrating stress signaling. Non-fluctuating concentrations of existing intracellular monosaccharides and disaccharides (soluble sugars like glucose and sucrose) (Katny et al., 2005; Zou et al., 2007)and increased accumulation of storage polysaccharides (starch) (Poorter et al., 1997) under elevated CO_2_ remain a mystery which can be taken up as future research to establish the link between ABA-signaling and intracellular starch proportion of the cell under elevated CO_2_ situation.

## 4 Conclusion

From the discussion of the current overview of the recent study, it can be concluded that abiotic stresses, particularly drought, high salinity, heavy metal, photo-stress, and high and low temperatures, are the major hindrances that limit crop productivity. After overviewing various earlier studies, the current study revealed that Zn-finger motifs have a significant role in the better understanding of abiotic stress. The study also recognized that a wide range of Zn-binding motifs, termed Zn-fingers’ proteins, had been identified. However, the function of stress-adaptation Zn-finger motifs is fully controlled by various genes. Speaking of abiotic stress and illustration the role of ZFP in wide range of plants, drought, salinity, temperatures etc. seems to be predominated. However, the involvements of ZFP in heavy metal stress are comparatively less than the other stresses. Moreover, the consequences of radio-nucleotides exposure to plants and the behaviour of ZFs will be an area of interest in the near future, as there is hardly any article addressing this issue. On the other hand, there is an ample scope to work on the effect of HM on the yield of crops as associated with various zinc finger protein. Another area of interest will be the documentation of the role of ZFP when plants were exposed to multiple stresses in different magnitudes. The information on the concept, importance, and mechanisms of Zn-finger motifs during abiotic stress response in plants will be helpful for the sustainability of crop production in the modern era of the changing climate.

## Author contributions

Conceptualization, DM, KB, BP, SM, TS, MS, UM, MB, and AH; writing—original draft preparation, DM, UM, SS, AR, KB, BP, SM, TS, MS, MB, VB, and AH; writing—review and editing, MB, MS, VB, SM, SH, and AH; Funding, MB, VB, MS, SM, and AH; All authors have read and agreed to submit the manuscript in Front. Plant Sci.
